# The Ophthalmic Branch of the Gutenberg Health Study: Study Design, Cohort Profile and Self-Reported Diseases

**DOI:** 10.1371/journal.pone.0120476

**Published:** 2015-03-16

**Authors:** René Höhn, Ulrike Kottler, Tunde Peto, Maria Blettner, Thomas Münzel, Stefan Blankenberg, Karl J. Lackner, Manfred Beutel, Philipp S. Wild, Norbert Pfeiffer

**Affiliations:** 1 Department of Ophthalmology, University Medical Center Mainz, Mainz, Germany; 2 Klinik Pallas, Olten, Switzerland; 3 NIHR Biomedical Research Center at Moorfields Eye Hospital, NHS Foundation Trust, and UCL Institute of Ophthalmology, London, United Kingdom; 4 Institute for Medical Biostatistics, Epidemiology and Informatics, University Medical Center Mainz, Mainz, Germany; 5 Preventive Cardiology and Preventive Medicine / Department of Medicine 2, University Medical Center Mainz, Mainz, Germany; 6 Clinic for General and Interventional Cardiology, University Medical Center Hamburg-Eppendorf, Hamburg, Germany; 7 Institute for Clinical Chemistry and Laboratory Medicine, University Medical Center Mainz, Mainz, Germany; 8 Department of Psychosomatic Medicine and Psychotherapy, University Medical Center Mainz, Mainz, Germany; 9 Center for Thrombosis and Hemostasis, University Medical Center Mainz, Mainz, Germany; 10 German Center for Cardiovascular Research (DZHK), partner site Rhine-Main, Mainz, Germany; Oregon Health & Science University, UNITED STATES

## Abstract

**Purpose:**

This paper describes the study design, methodology, cohort profile and self-reported diseases in the ophthalmological branch of the Gutenberg Health Study (GHS).

**Methods:**

The GHS is an ongoing, prospective, interdisciplinary, single-center, population-based cohort study in Germany. The main goals of the ophthalmological section are to assess the prevalence and incidence of ocular diseases and to explore risk factors, genetic determinants and associations with systemic diseases and conditions. The eye examination at baseline included a medical history, self-reported eye diseases, visual acuity, refractive errors, intraocular pressure, visual field, pachymetry, keratometry, fundus photography and tear sampling. The 5-year follow-up visit additionally encompassed optical coherence tomography, anterior segment imaging and optical biometry. The general examination included anthropometry; blood pressure measurement; carotid artery ultrasound; electrocardiogram; echocardiography; spirometry; cognitive tests; questionnaires; assessment of mental conditions; and DNA, RNA, blood and urine sampling.

**Results:**

Of 15,010 participants (aged 35-74 years at the time of inclusion), ocular data are available for 14,700 subjects (97.9%). The mean visual acuity (standard deviation), mean spherical equivalent, median decimal visual acuity, and mean intraocular pressure were 0.08 (0.17) logMar, -0.42 (2.43) diopters, 0.9 and 14.24 (2.79) mm Hg, respectively. The frequencies of self-reported strabismus, glaucoma, surgery for retinal detachment and retinal vascular occlusions were 2.7%, 2.3%, 0.2% and 0.4%, respectively.

**Conclusions:**

The GHS is the most extensive dataset of ophthalmic diseases and conditions and their risk factors in Germany and one of the largest cohorts worldwide. This dataset will provide new insight in the epidemiology of ophthalmic diseases and related medical specialties.

## Introduction

Several epidemiological studies have reported the prevalence, incidence and risk factors of major ophthalmic diseases. However, there may be large differences between populations and even within the same population at different points in time [1]. This variability might be due to demographic differences or genetic and environmental factors, or it might be partly explained by methodological differences [2, 3].

Knowledge of the precise epidemiological features of a population is essential for planning the future use of medical resources [4]. As population age and environmental risk factors change, thorough epidemiological research is necessary to acquire the knowledge to ensure sufficient medical care, public health screenings and the efficient use of medical resources.

Several major ophthalmological diseases are linked to systemic diseases such as atherosclerosis, diabetes mellitus or arterial hypertension. Changes in the prevalence, incidence and treatment of those systemic risk factors will change the occurrence and course of the associated ophthalmological diseases. Furthermore, there is eminent need to investigate the association between eye diseases and related cardiovascular, neurodegenerative, metabolic and immunological conditions.

The ophthalmological branch of the Gutenberg Health Study (GHS) was designed and established to

- estimate the prevalence and incidence of major ocular diseases (such as glaucoma, age-related macular degeneration, diabetic retinopathy) and their risk factors in Germany, which is the European country with the largest population,
- report distribution on biometric ocular parameters within a population-based study design,
- provide data on associations between ocular parameters (e.g. intraocular pressure, refractive error, central retinal thickness) and diseases (e.g. diabetic retinopathy, age-related macular degeneration, glaucoma) with in particular cardiovascular parameters and diseases, genetic variants, mental and general conditions,
- establish a large biobank for future research projects.

This article describes the study structure and methods of the ophthalmic branch of the GHS, presents its other major parts and research topics and provides data on the cohort profile and self-reported ocular diseases.

## Materials and Methods

The Gutenberg Health Study (GHS) is an ongoing population-based, interdisciplinary, prospective, single-center cohort study at the medical center of Johannes Gutenberg University Mainz in Germany. All participants provided informed consent. The study design followed the tenets of the Declaration of Helsinki. The GHS was approved by the local ethics committee (Ethics Commission of the State Chamber of Physicians of Rhineland-Palatinate) and by the local and federal data safety commissioners.

### Study aim

The GHS aims to assess the prevalence and incidence of cardiovascular, metabolic, ophthalmological and mental disorders in the population of the city of Mainz and the Mainz-Bingen district in the mid-western part of Germany (Rhine-Main region). The primary goal of the study is to improve the prediction of disease by taking into account psychosocial, environmental and lifestyle risk factors, subclinical disease, protein pattern and genetic variants. The sample size calculation was based on the primary endpoints of the study, myocardial infarction and cardiovascular death.

### Study design

A pilot study of feasibility and for establishing process sequences was conducted between 2005 and 2007. The baseline assessment of the population-based cohort of 15,010 men and women aged 35 to 74 at the time of inclusion started in April 2007 and was completed in March 2012. An interdisciplinary examination protocol was applied, including general, anthropometric, cardiovascular, psychological and ophthalmological tests lasting 5 hours. A computer-assisted interview and questionnaires elucidated the classic psychosocial, environmental, physical activity, nutritional and lifestyle risk factors. Two and a half years after the baseline examination, the participants were contacted for a follow-up investigation by computer-assisted telephone interview, which recorded the end-points, medical history and self-reported medication and lifestyle factors. Five years after the baseline visit, the participants are invited to the study center for a follow-up visit, comprising of repeated measurements with an extended examination protocol lasting nearly 6 hours. A second telephone interview will take place 7.5 years after the baseline visit. Furthermore, a 10-year follow-up of the whole cohort is planned commencing in 2017.

### Study population

Out of all residents of the city of Mainz (n = 196,425) and the district of Mainz-Bingen (n = 201,371) 210,867 subjects aged 35 to 74 years at the time of inclusion were eligible to participate in the GHS. Of these, a sample of 35,008 subjects, stratified by gender, decade of age and residence (rural vs. urban), was selected via the local residents’ registration offices. A study sample of intended 15,000 participants was drawn in waves of equal stratification to meet a standardized recruiting and to allow defined subsample analyses after inclusion of 5,000 and 10,000 subjects. The exclusion criteria were insufficient knowledge of the German language and physical or mental inability to participate in the examinations at the study center. A total of 15,010 subjects were enrolled in the study sample and the overall response rate was 60.3%.

### Enrollment methods

The study participants were selected randomly from local residents’ registration offices. According to German law, it is mandatory for each individual to register his/her personal and residential data within a week after moving to or from any location within Germany. Selected participants were contacted by letter and invited for the baseline examination. If they did not respond, then the recruitment team contacted them by phone. Persons, who were unable to come to the study center or did not want to participate, were asked to respond to some questions about demographics and their reasons for non-participation (non-responder questionnaire). A staged consent process was established for non-invasive testing, blood sampling and genetic assessments. The participants could withdraw from the study at any point without a need for justification. Consenting withdrawal immediately resulted in disposal of all bio-samples.

### Data management / Steering committee

The data were collected, managed and analyzed at a central data bank under the supervision and responsibility of the GHS study coordinator. Plausibility checks and data quality controls were performed on a regular basis. The GHS steering committee decided on the internal and external access and use of the data and biomaterials.

### General and non-ophthalmological tests

All data acquisition and tests were performed in a standardized manner by trained and certified personnel at the GHS study center. The sequence of examinations and interviews was predetermined. Table 1 gives an overview of non-ophthalmological tests, questionnaires and interviews.

**Table 1:** Summary of non-ophthalmological tests, questionnaires and interviews of the Gutenberg Health Study (baseline examination).

**General data**	Anthropometry
	Body temperature
	Current weather data
	Physical activity
	Nutrition
	Medications
**Cardiology, angiology and pulmonology tests**	Electrocardiography
	Echocardiography
	Blood pressure and pulse
	Sonography of neck vessels
	Neurocardiac regulation
	Occlusion pressure measurement and ankle brachial index
	Spirometry
	Flow-mediated vasodilation and arterial stiffness, volume plethysmography of finger artery, digital photo plethysmographical pulse curve analysis
	Carbon monoxide in alveolar air
**Laboratory tests**	Blood count, electrolytes, renal and liver function parameters, blood fat parameters
	Basic and special blood coagulation parameters, cardiac enzymes
	Inflammatory parameters, selected vitamins and hormones
	Parameters of oxidative stress, basic urine tests
**Psychological and social data**	Social demographic data
	Access to and use of medical care
	Cancer prevention
	Gender-related questions
	Full medical history
	Classic cardiovascular risk factors
	Disease-related complaints and pathologies
	Family medical history
	Children
	Health care behavior
	Hobbies
	Smoking, passive smoking, alcohol consumption
	Occupational history
	Exposure to airborne pollutants and noise
	General happiness and environmental factors
	Domestic environment
	Neurocognitive function
	Personality, psychiatric diseases and mental disorders
	Everyday duties
	Social integration
	Psycho-social stress at work
	Life events
	Visual quality of life
**Biomaterials**	Plasma, serum, DNA, RNA, urine, gingival sulcus swab, lacrimal fluid

### Ophthalmological examinations

Table 2 summarizes the ophthalmological examinations of the GHS, which lasted 25 minutes per participant and were performed between 11 a.m. and 8 p.m. During the baseline visit all investigations were performed by a board-certified ophthalmologist. Starting with the 5-year follow-up in April 2012, all examinations were being performed by well-trained and certified study assistants. The study protocol was modified with the follow-up phase; all changes are shown in Table 2.

**Table 2:** Gutenberg Health Study eye examinations at baseline (2007–2012) and at the 5-year follow-up (2012–2017).

	Manufacturer/Methods/Specifications	
	*Baseline*	*Follow-up*
**Autorefraction**	Humphrey Automated Refractor/Keratometer (HARK) 599^1^	
**Corrected visual acuity**		
**Pachy- and keratometry**	Pachycam^2^	Pentacam^2^
**Visual field screening**	FDT Humphrey Matrix Perimeter^1^	
**Intraocular pressure**	Nidek NT-2000 Noncontact tonometer^3^	
**Biometry**	-	Lenstar LS 900^4^
**Slit-lamp examination**	Haag-Streit BM 900^4^	-
**Fundus photography**	Visucam ^*PRO NM*,^ ^1^ 45° field fundus, 30° field optic disc and macular area	
**Optical Coherence Tomography**	Spectralis-OCT^5^, ^6^	Spectralis-OCT^5^
**Schirmer II Tear Test^7^/ collection of tear fluid**	Oxybuprocaine-hydrochloride 0,4% eye drop^8^, Schirmer Tear Test Ophthalmic Strips^9^	

^1^Carl Zeiss Meditec AG, Jena, Germany

^2^Oculus, Wetzlar, Germany

^3^Nidek Co., Japan

^4^Haag Streit, Koeniz, Switzerland

^5^Heidelberg Engineering, Heidelberg, Germany

^6^part of the examination since 2011

^7^performed only at baseline visit

^8^OmniVision, Puchheim, Germany

^9^Optitech Eyecare, Allahabad, India,

#### Refraction and Visual acuity

Autorefraction and corrected visual acuity were measured using a Humphrey Automated Refractor / Keratometer (HARK) 599 (Carl Zeiss AG, Jena, Germany). The right eye was measured first. After obtaining autorefraction, corrected visual acuity was recorded using the built-in Snellen charts, ranging from 20/400 to 40/20 (decimal 0.05 to 2.0). If the visual acuity was below 20/400 (decimal 0.05), further testing was performed using a visual acuity chart at a distance of one meter up to 20/800, and further down to counting fingers, hand movements, light perception and no light perception at the lowest level. The spherical equivalent was calculated as the spherical correction value plus half the cylindrical power.

#### Intraocular pressure

Intraocular pressure was measured with a noncontact tonometer and automatic air-puff control (Nidek NT-2000; Nidek, Co., Gamagori, Japan). Always starting with the right eye, the mean of three measurements within a 3-mmHg range was obtained for each eye.

#### Visual field testing

Frequency doubling technology (FDT) Humphrey Matrix Perimeter was used for visual field testing (program N-30-5). The participants were asked whether they had any experience with visual field testing (yes/no) and started the test with their right eye, while those with an odd study ID number started with the left eye. To allow the participant to relax, the measurement of corneal thickness (described below) was performed in between the visual field testing of the first and the second eye. Subjects with a refractive error between +5.0 and -5.0 diopters spherical equivalent underwent the examination without correction; subjects with a refractive error higher or lower than +5.0 or -5.0 diopters spherical equivalent used their corrective devices (glasses or contact lenses). If defects with a threshold of either one abnormal cluster/field with *P*<1% or two adjacent clusters/fields with *P*<5% became apparent, the examination was immediately repeated for the affected eye. The examination result was promptly classified as a) normal or b) conspicuous.

#### Pachymetry / Keratometry

A noncontact, Scheimpflug-based optical device (Pachycam, Oculus, Wetzlar, Germany) was used to measure the corneal thickness and curvature during the baseline examination of all participants. Only measurements with a quality score above 90% were accepted. Pachymetry is based on Scheimpflug images of a horizontal 4-mm section and reveals the central and thinnest corneal thicknesses. The Pachycam has an integrated keratometer that measures the central K-value as well as the K-values in the central 30°. Beginning with the 5-year follow-up, we replaced the Pachycam with the Pentacam HR (Oculus, Wetzlar, Germany) for corneal thickness measurements and keratometry. The Pentacam uses the same measurement technology, but it provides additional data on the architecture of the anterior segment of the eye (see anterior segment tomography). All of these tests started with the right eye.

#### Posterior segment photography

A Visucam ^*PRO NM*^ nonmydriatic fundus camera (Carl Zeiss AG, Jena, Germany) was used to take three digital fundus pictures through a non-pharmacologically dilated pupil. The participants were positioned in a darkened room to allow for natural pupil dilation in preparation for posterior segment photography. Two images were centered on the optic disc (30° and 45° field) and one on the macula (30°). We always started with the right eye. At baseline, the ophthalmologist conducted a simple evaluation of the fundus pictures according to the parameters listed in Table 3.

**Table 3:** Summary of recorded ophthalmic parameters in the electronic case report file (eCRF, baseline examination).

**Oculomotoric characteristics**	Eye position according to corneal reflexes
	Heterotropia related to nerve palsy
**Eyelid**	Grading of blepharitis (normal/mild/moderate/severe)
	Lid position (normal/entropion/ectropion)
**Anterior segment**	Conjunctiva (hyperemia, LIPCOF, pinguecula)
	Cornea (general pathologies)
	Anterior chamber (cells, flare)
	Iris (color, atrophic areas, pigment dispersion, rubeosis, synechiae)
	Lens (status, cataract, secondary cataract, pseudoexfoliation)
**Posterior segment**	Pathologies in general (yes/no)
	Cone at the optic disc (yes/no)
	Glaucomatous disc atrophy (no/suspicious/beginning/advanced)
	Other optic nerve atrophies (yes/no)
	Hypertensive retinopathy (grading according to Keith-Wagener-Barker classification [32])
	Macular drusen (yes/no)
	Age-related macular degeneration (no/dry/geographic atrophy/wet/scar)
	Diabetic retinopathy (no/mild/moderate/proliferative)
	Retinal vein occlusion (no/branch/hemi-central/central)
	Retinal arterial occlusion (no/branch/hemi-central/central)
	Retinal detachment (yes/no)

#### Slit-lamp biomicroscopy of the anterior segment

A slit-lamp examination was performed with undilated pupils in a darkened room to detect pathologies of the anterior segment. Table 3 provides a summary of recorded parameters and conditions.

#### Anterior segment tomography

Non-contact anterior segment tomography was introduced to the study protocol in April 2012 as the 5-year follow-up testing commenced. The Pentacam HR (Oculus, Wetzlar, Germany) uses an automatically rotating Scheimpflug camera to measure anterior segment architecture, including the entire cornea, anterior chamber and lens. The Pentacam provides data on the following parameters: central corneal radii, corneal thickness and asphericity, colored maps of curvature and elevation, chamber angle, volume and elevation, as well as lens transparency. Pharmacological pupil dilation was not applied. Photo documentation was performed on the right eye first.

#### Ocular biometry

Non-contact optical biometry was added to the study protocol in April 2012, commencing with the 5-year follow-up. Lenstar LS900 (Haag-Streit, Koeniz, Switzerland) employs optical low-coherence reflectometry technology to capture the ocular globe's axial dimensions. A single measurement yields data on the following parameters: central corneal thickness, keratometry, white-to-white corneal distance, pupil diameter (in darkened room), lens thickness, anterior chamber depth, the ocular globe's axial length, visual axis eccentricity and retinal thickness (distance between internal limiting membrane and retinal pigment epithelium). The right eye was measured first.

#### Optical coherence tomography (OCT)

Spectral domain optical coherence tomography was performed with the SPECTRALIS (Heidelberg Engineering, Heidelberg, Germany). The scans were taken through non-pharmacologically dilated pupils in the darkened examination room. One macular volume scan (a modified posterior pole scan) with enhanced depth imaging (EDI) and two optic disc scans—an optic nerve head volume scan with EDI and a peripapillary retinal nerve fiber layer (RNFL) scan—were conducted.

#### Tear sampling / Biobanking

Tear fluid samples were taken from both eyes of each participant using Schirmer’s Tear Ophthalmic strips (Optitech Eyecare, Allahabad, India) one minute after application of local anesthetic drops of Oxybuprocaine-hydrochloride 0.4% (OmniVision, Puchheim, Germany). The strips were placed in the lower conjunctival sac for three minutes. If the strip became saturated before the scheduled end of the test, it was immediately removed from the conjunctival sac. At the baseline visit, the ophthalmologist also classified the amount of fluid into three categories a) normal, b) slightly reduced or c) severely reduced tear production. As all biomaterials, tear samples are stored at -80°C in two segregated rooms using a standardized and partially automatized storing system. The temperature is monitored electronically.

#### Visual Functioning Questionnaire and family history of age-related macular degeneration and glaucoma

The German version of the “National Eye Institute Visual Functioning Questionnaire 25” (NEI VFQ-25) was used [5]. During the computer-assisted personal interview (CAPI), all participants were asked whether they were aware of any age-related macular degeneration or glaucoma in their family history (parents or siblings).

### Self-reported eye diseases

Self-reported eye diseases were extracted from the patient history collected along with the eye examination. Every participant was asked if they suffer from any kind of glaucoma, macular degeneration or any other eye disease. The diseases were clustered to their clinical and colloquial terms and analysed respectively.

### Classification of major eye diseases

The definitions to detect glaucoma [6], age-related macular degeneration [7, 8] and diabetic retinopathy [9] are listed in Table 4.

**Table 4:** Definition of glaucoma, age-related macular degeneration and diabetic retinopathy in the Gutenberg Health Study.

Disease	Definition
**Glaucoma**	ISGEO glaucoma classification [6]
**Age-related Macular Degeneration**	Rotterdam Eye Study classification [7, 8]
**Diabetic Retinopathy**	Early Treatment Diabetic Retinopathy Study (ETDRS) [9]

### Genetic analyses

Genomic DNA extraction was obtained from two buffy-coated EDTA blood samples from each participant. The DNA was extracted according to Miller's method [10]. DNA samples from the first 5000 GHS participants were genotyped using the Affymetrix GeneChip Genome-Wide Human SNP Array 6.0 in batches of 94 GHS samples, including one reference genomic DNA by Affymetrix (Ref. 103, 50 ng/μl) and one negative control without genomic DNA per batch[11]. Total RNA was isolated from monocytes (first 1700 participants) and peripheral blood mononuclear cells (all remaining participants). RNA was isolated only for those participants presenting at the study center during the morning session.

RNA was isolated within 24 h after blood sampling to ensure rapid sample processing. Monocytes were separated from whole blood, and RNA was prepared as described previously [11]. The integrity of the entire RNA was assessed through analysis on an Agilent Bioanalyzer 2100 (Agilent Technologies, Böblingen, Germany). Samples with an RNA-Integrity number (RIN) less than seven were excluded from further analyses.

Transcriptome analyses of RNA were performed for 2200 participants using an Illumina Ht-12 BeadChip V3 (n = 1 700) and the Affymetrix GeneChip Human Exon 1.0 ST Array (n = 500) [11, 12]. Further genetic testing will be possible using the stored biosamples. We plan to use next-generation sequencing approaches for further genetic analyses of the DNA and RNA samples. Furthermore, sequencing of miRNA in the total RNA samples as well as epigenetic assessments will be performed.

### Quality assurance

All study-related activities were performed according to “Good Clinical Practice”, “Good Epidemiological Practice” and the tenets of the declaration of Helsinki. Standard operating procedures (SOPs) were available for all tests, and the sequence of study-related activities was predetermined and is binding. The SOPs were verified and set during the pilot phase of the study and between the end of the baseline examination and the beginning of the 5-year follow-up. A central data management team is in charge of performing plausibility tests and descriptive statistics on a regular basis.

### Cardiovascular risk factor assessment

Cardiovascular risk factors (CVRFs) were defined as follows: Smoking was dichotomized into non-smokers (never smokers and ex-smokers) and smokers (occasional smokers and smokers). Arterial hypertension was diagnosed if antihypertensive drugs were taken, if the mean systolic blood pressure was ≥140 mm Hg in the 2^nd^ and 3^rd^ standardized measurement after 8 and 11 minutes of rest or if the mean diastolic blood pressure was ≥90 mm Hg in the 2^nd^ and 3^rd^ standardized measurement after 8 and 11 minutes of rest. Diabetes was stated for individuals, who at least hold one of the following conditions: (a) definite diagnosis and treatment of diabetes by a physician, (b) a blood glucose level ≥126 mg/dl at the baseline examination after overnight fasting of at least 8 hours or (c) a blood glucose level of ≥200 mg/dl at the baseline examination after a fasting period of at least 8 hours. Obesity was determined as a body mass index (BMI) ≥30 kg/m². Dyslipidemia was defined as a definite diagnosis of dyslipidemia by a physician or a LDL/HDL-ratio of ≥3.5. Anthropometric measurements were performed with calibrated digital scales (Seca 862, Seca, Hamburg Germany), a measuring stick (Seca 220, Seca, Hamburg, Germany) and a waist-measuring tape.

### Self-reported cardiovascular diseases

Self-reported cardiovascular disease data were collected from computer-assisted personal interviews.

### Statistical analyses

All data underwent quality control by a central data management team and were checked for completeness and correctness by predefined algorithms and quality plausibility controls. Prevalences, rates and mean values are always presented for the study sample (no special indication, only in tables indicated as “unweighted”) and weighted for the local population in the city of Mainz and the Mainz-Bingen district (indicated as “weighted”) because the older age decade was overrepresented in our study sample. Weighted data are necessary to provide population-based results and to achieve comparability to other population-based data.

## Results

After completing a pre-study phase, the study began in 2007, and the baseline examination was completed in March 2012. The 5-year follow-up of the cohort started in April 2012. The GHS cohort comprised a total of 15,010 participants.

Of these, 14,700 participants or 97.9% of the whole study sample attended the eye examination. Due to unavailability of ophthalmological examining personnel (e.g. sickness leave), 310 attendees did not undergo the eye examination. The age distribution of the examined and non-examined subjects is presented in Tables 5 and 6, respectively. The participants’ mean age, the weighted mean age and the non-examined subjects mean age was 55.0 (standard deviation ±11.1, range 35–74) years, 52.6 (±11.1) years and 56.3 (±10.8) years, respectively. Men were slightly overrepresented, with 7,420 men (50.5%) versus 7,282 women (49.5%), weighted as 7,317.3 (49.8%) and 7,382.7 (50.2%), respectively.

**Table 5:** Age distribution of participants with ophthalmological examinations in the Gutenberg Health Study at baseline (2007–2012), for the study sample and weighted for the population of Mainz/ Mainz-Bingen.

Age range (years)	Male, *n* (%)		Female, *n* (%)		Total, *n* (%)	
	Sample	Weighted	Sample	Weighted	Sample	Weighted
**35–44**	1556 (10.6%)	2185.1 (14.9%)	1671 (11.4%)	2111.2 (14.4%)	3227 (22.0%)	4296.4 (29.2%)
**45–54**	1986 (13.5%)	2198.4 (15.0%)	1941 (13.2%)	2172.4 (14.8%)	3927 (26.7%)	4370.8 (29.7%)
**55–64**	1973 (13.4%)	1561.5 (10.6%)	1908 (13.0%)	1589.1 (10.8%)	3881 (26.4%)	3150.5 (21.4%)
**65–74**	1905 (13.0%)	1372.4 (9.3%)	1760 (12.0%)	1510.0 (10.2%)	3667 (24.9%)	2882.3 (19.6%)
**All ages**	7420 (50.5%)	7317.3 (49.8%)	7282 (49.5%)	7382.7 (50.2%)	14700 100%)	14700 100%)

**Table 6:** Age distribution of participants without ophthalmological examination in the Gutenberg Health Study at baseline (2007–2012).

Age range (years)	Male, *n* (%)	Female, *n* (%)	Total, *n* (%)
**35–44**	26 (8,4%)	33 (10.7%)	59 (22.0%)
**45–54**	28 (9.0%)	32 (10.3%)	60 (19.4%)
**55–64**	60 (19.4%)	43 (13.9%)	103 (33.2%)
**65–74**	50 (16.1%)	38 (12.3%)	88 (24.9%)
**All ages**	164 (52.9%)	146 (47.1%)	310 (100%)

The prevalence of arterial hypertension was 49.6% (weighted prevalence 45.1%; non-examined subjects 58.1%), diabetes mellitus 7.5% (6.3%; 8.8%), obesity 25.2% (24.2%; 25.4%), and of smoking 19.4% (20.7%; 19.7%) among the participants attending the eye examination.

A summary of the ocular characteristics of the investigated participants is shown in Table 7. The major findings were a mean visual acuity of 0.08 logMar (weighted 0.07) and a mean spherical equivalent, calculated as the sphere plus half of the cylinder, of -0.42 ± 2.43 diopters (weighted -0.56 ± 2.44 diopters). Median decimal visual acuity was 0.9 (weighted 1.0). The mean intraocular pressure was 14.24 ± 2.79 mmHg (weighted, 14.21 ± 2.78 mmHg). Approximately 90% of the participants said that they used some type of glasses, and approximately 76% used sunglasses.

**Table 7:** Ocular characteristics of participants in the Gutenberg Health Study at baseline (2007–2012), for the study sample and weighted for the population of Mainz/ Mainz-Bingen.

	*n* ^*1*^	Sample	Weighted
**Visual acuity^**2**^ (logMar)**	*14699*	0.08 (0.17) [0.08–0.09]	0.07 (0.16) [0.07–0.08]
**Spherical equivalent^**2**^ (diopter)**	*14452*	-0.42 (2.43) [-0.46- (-0.38)]	-0.56 (2.44) [-0.60- (-0.52)]
**Intraocular pressure^**2**^ (mm Hg)**	*14587*	14.24 (2.79) [14.20–14.29]	14.21 (2.78) [14.17–14.26]
**Wearing glasses, n (%) [CI]**	*14700*	13119 (89.2%) [88.8%-89.7%]	12677.6 (86.2%) [85.6%-86.9%]
**Wearing sunglasses, n (%) [CI]**	*14700*	11099 (75.5%) [74.8%-76.2]	11429.1 (77.8%) [77.1%-78.4%]
**Ocular medication, n (%) [CI]**	*14700*	1811 (12.3%) [11.8%-12.8%]	1640.9 (11.2%) [10.7%-11.7%]
**History of ocular surgery, n (%) [CI]**	*14700*	1141 (7.8%) [7.3%-8.2%]	1005.8 (6.8%) [6.4%-7.2%]
***Self-reported eye diseases*** ^***3***^			
**Cataract, n (%) [CI]**	14700	97 (0.66%) [0.54%-0.80%]	84.8 (0.58%) [0.46%-0.69%]
**Glaucoma, n (%) [CI]**	14700	331 (2.3%) [2.0%-2.5%]	282.5 (1.9%) [1.7%-2.1%]
**Suspected glaucoma, n (%) [CI]**	14700	8 (0.05%) [0.02%-0.10%]	6.9 (0.05%) [0.01%-0.08%]
**Ocular hypertension, n (%) [CI]**	14700	27 (0.18%) [0.12%-0.26%]	23.3 (0.16%) [0.10%-0.22%]
**Age-related macular degeneration, n (%) [CI]**	14700	69 (0.47%) [0.36%-0.59%]	55.0 (0.37%) [0.29%-0.46%]
**Retinal detachment, n (%) [CI]**	14700	27 (0.18%) [0.12%-0.26%]	22.1 (0.15%) [0.09*%-0.21%]
**Retinal tear, n (%) [CI]**	14700	26 (0.18%) [0.12%-0.25%]	24.5 (0.17%) [0.10%-0.23%]
**Retinal vessel occlusion, n (%) [CI]**	14700	51 (0.35%) [0.26%-0.44%]	43.1 (0.29%) [0.21%-0.38%]
**Uveitis, n (%) [CI]**	14700	71 (0.48%) [0.37%-0.60%]	75.8 (0.52%) [0.39%-0.64%]
**Strabismus, n (%) [CI]**	14700	395 (2.69%) [2.41%-2.94]	413.6 (2.81%) [2.53%-3.10%]
**Traumatic Eye disease, n (%) [CI]**	14700	122 (0.83%) [0.69%-0.98%]	119.8 (0.82%) [0.67%-0.96%]
**Dry eye syndrome, n (%) [CI]**	14700	236 (1.61%) [1.39%-1.80%]	208.4 (1.42%) [1.23%-1.60%]
**Color deficiency, n (%) [CI]**	14700	24 (0.16%) [0.10%-0.24%]	25.5 (0.17%) [0.10%-0.25%]
**Optic Neuritis, n (%) [CI]**	14700	20 (0.14%) [0.07%-0.20%]	19.8 (0.13%) [0.07%-0.20%]
**Allergic conjunctivitis, n (%) [CI]**	14700	91 (0.62%) [0.50%-0.76%]	91.7 (0.62%) [0.49%-0.76%]
**Retinitis pigmentosa, n (%) [CI]**	14700	7 (0.05%) [0.02%-0.09%]	6.0 (0.04%) [0.01%-0.07%]
**Nystagmus, n (%) [CI]**	14700	17 (0.12%) [0.07%-0.18%]	19.8 (0.13%) [0.07%-0.20%]

n = number of subjects, CI = confidence interval

^1^ *n* varies between 14452 and 14700 due to missing data.

^2^Values are indicated as mean (standard deviation) [confidence interval].

^3^Extracted from medical history.

Information on the ophthalmological history and self-reported eye diseases was available for 14,700 subjects (Table 7). Approximately 12% of the participants used ocular medication, and approximately 8% had a history of ocular surgery.

The frequency of self-reported eye diseases was 2.3% for glaucoma, 0.2% for surgery for retinal detachment, approximately 0.4% for retinal vascular occlusions, 2.7% for strabismus, 0.12% for nystagmus and 0.05% for retinitis pigmentosa. A history of ocular trauma was reported by 0.8% of the participants.

## Discussion

Reliable epidemiologic data are necessary to develop prevention strategies and to plan for the future use of medical resources in a population. Few population-based studies have examined the extent of visual impairment and ocular health in Germany, the European country with the largest population. The Study of Health in Pomerania (SHIP) will be able to provide only few data because their ophthalmological examination is limited to retinal photography [13]. The Cooperative Health Research in the Region of Augsburg (KORA) Eye study is based on a standardized interview and an eye disease questionnaire administered to 2,593 participants, with partial validation through the treating ophthalmologist. The KORA Eye Study provides data on only four self-reported eye diseases (corneal disease, cataracts, retinal disease and glaucoma [14]). The Leipzig Research Center for Civilization Diseases Study (Leipziger Forschungszentrum für Zivilisationserkrankungen, LIFE) is another large cohort in the eastern part of Germany; however, the acquisition of ophthalmic data is limited to fundus photography and optical coherence tomography. Thus, there was a genuine need for a comprehensive, population-based epidemiological study of ocular diseases in Germany.

The ophthalmic branch of the GHS was designed to investigate the prevalence, incidence and relevant risk factors associated with major ophthalmic diseases and conditions in a predominantly Caucasian population [15, 16]. Additional main goals of the study are to provide normative data, to assess the genetic basis and associations between ophthalmological parameters and diseases and to provide a biobank for future research.

The main strengths of the GHS are 1) its population-based design, 2) the multidisciplinary approach, 3) the large size of the cohort, 4) longitudinal data acquisition and 5) genotyping in addition to meticulous ophthalmic phenotype determination. The protocol allows us to investigate the association between eye parameters and diseases in reference to a vast number of risk factors and general health conditions, including general anthropometric data, cardiovascular risk factors, laboratory parameters, socioeconomic status and mental health factors.

The entire sample and the first 5,000 and 10,000 participants are stratified by gender, age (by decade) and residence (urban vs. rural); thus, any new hypothesis generated within a certain sub-cohort can be examined in the remaining sub-cohorts. Stratification by age has led to over-representation of the elderly in the GHS cohort. The overrepresentation of the higher age groups is intended to ensure a sufficient number of probable cardiovascular events and other age-related diseases for statistical analysis. Therefore, the reported prevalence is standardized for the study population (Mainz / Mainz-Bingen). This age distribution is of particular relevance to ocular diseases, as most of these diseases are more prevalent in the elderly. The upper age at baseline is limited to 74 years. Including persons who were older than 75 would have been beneficial for assessing most age-related eye diseases and conditions. We opted to set the age limit at the mid-seventies at baseline to ensure greater likelihood of a high percentage of re-participation at the 5- and 10-year follow-ups.

The GHS cohort is stratified by the residences of the participants, distinguishing between residents of the large city of Mainz and rural residents living in the Mainz-Bingen district. When considering the difference between these two groups, one should be aware that the city and district are very close to each other geographically. Furthermore, the recruitment area of the study is located within the densely populated and highly industrialized region of Rhine-Main, which is why we do not anticipate large differences between urban and rural participants, as previously reported by studies in other countries [17, 18].

Our study design, particularly the limited time for the eye examination, creates limitations. The entire eye examination is carried out in pharmacologically unaffected pupils, which jeopardizes, for example, thorough categorization of cataract stages or the detection of pseudo-exfoliation, as well as evaluation of the retinal periphery. The slit-lamp examination does not include characterization of the iridocorneal angle and Goldmann applanation tonometry.

We have previously published results on intraocular pressure (IOP) [19]; these findings revealed a lower mean IOP than in other Caucasian samples, but similarly positive associations between the central corneal thickness and cardiovascular risk factors. Surprisingly, we observed a negative association between age in women and IOP.

The GHS has the potential to provide insight into numerous interdisciplinary relationships between ocular and systemic diseases. One example is the assessment of the predictive value of diabetic retinopathy for the future development of cardiac failure, as reported by a Japanese group [20].

Collecting self-reported data on eye diseases is a simple method for gathering prevalence data. However, many study participants are unaware of eye diseases, especially at early stages (e.g., early glaucoma, mild age-related macular degeneration or mild cataract) [21, 22]. This lack of awareness is exemplified by our analysis of age-related macular degeneration (AMD), in which we evaluated fundus pictures of the first 5,000 participants. Signs of early AMD were detected in 11.9% of the overall sample and in 3.8% of the youngest sample group (aged 35–44) [7]. The self-reported overall prevalence rate was approximately 0.5%, which is dramatically lower than the graded prevalence. Thus, self-reported data should be validated against physician-confirmed diagnoses, eye examinations or in particular, epidemiological studies applying standardized reading procedures (e.g. reading centers). Such grading processes are very time- and resource-consuming, which is why future developments in (semi-) automatic data interpretation will help to provide necessary data in an easier and more standardized way [23].

The self-reported prevalences of most diseases are, therefore, lower in our study than in other studies [21, 24]. However, regarding those diseases that are much more likely to decrease quality of life, we observed similar or even higher self-reported prevalences. Retinitis pigmentosa is reported to have a prevalence of approximately 0.025 [25]. The prevalence in our sample is 0.05% (7 of 14,700), which is twice the prevalence cited in the literature. The prevalence of nystagmus has been estimated at between 0.033 and 0.1 [26]. However, Sarvanathan et al. found an overall prevalence of 0.17% (17 of 10,000) in children up to 17 years old [27]. The self-reported prevalence of nystagmus in our adult cohort was 0.12% (17 of 14,700), which is considerably close to the estimated and measured values. We observed a strabismus coincidence (2.7%) similar to those reported in several cohorts of children (2.3–2.8%) [28–31].

Thus, self-reported eye diseases still play an important role in epidemiological studies both as exclusion criteria and to provide reliable prevalence data on eye diseases that disable or threaten quality of life.

In the GHS, there is also a considerable chance of underestimating the prevalence rates, particularly of blind and visually impaired people. This group might be underrepresented in our cohort due to a lower willingness to participate in a trial, which could directly result from their impairment. Furthermore a limited underestimation of prevalence rates might be through the subjects without eye examination. Although those participants are a small number (n = 310 or 2.1%), they are slightly older (65.3 vs. 55.0 years) and more affected by arterial hypertension (58.1% vs. 49.6%) and diabetes mellitus (8.8% vs. 7.5%) than the examined subjects.

The GHS will provide valuable data on diseases of the eye, their risk factors and associations, as well as their genetic background. Five-year incidence data will be available after the follow-up tests in the study's first 5,000 participants have been completed. The entire cohort will have completed the 5-year follow-up by March 2017. The steering committee of the GHS intends to prolong the study, with the 10-year follow-up commencing in 2017.
